# Dual Mode of Mitochondrial ROS Action during Reprogramming to Pluripotency

**DOI:** 10.3390/ijms231810924

**Published:** 2022-09-18

**Authors:** Elena V. Skvortsova, Igor B. Nazarov, Alexey N. Tomilin, Sergey A. Sinenko

**Affiliations:** Institute of Cytology, Russian Academy of Sciences, Tikhoretsky Ave. 4, 194064 St. Petersburg, Russia

**Keywords:** Ndufs1, Ndufb10, induced pluripotent stem cells (iPSCs), reactive oxygen species (ROS), rotenone, complex I (CI) of electron transport chain (ETC), hydrogen peroxide, superoxide anion

## Abstract

Essential changes in cell metabolism and redox signaling occur during the reprogramming of somatic cells into induced pluripotent stem cells (iPSCs). In this paper, using genetic and pharmacological approaches, we have investigated the role of electron transport chain (ETC) complex-I (CI) of mitochondria in the process of cell reprogramming to pluripotency. Knockdown of NADH-ubiquinone oxidoreductase core subunits S1 (Ndufs1) or subunit B10 (Ndufb10) of the CI or inhibition of this complex with rotenone during mouse embryonic fibroblast (MEF) reprogramming resulted in a significantly decreased number of induced pluripotent stem cells (iPSCs). We have found that mitochondria and ROS levels due course of the reprogramming tightly correlate with each other, both reaching peak by day 3 and significantly declining by day 10 of the process. The transient augmentation of mitochondrial reactive oxygen species (ROS) could be attenuated by antioxidant treatment, which ameliorated overall reprogramming. However, ROS scavenging after day 3 or during the entire course of reprogramming was suppressive for iPSC formation. The ROS scavenging within the CI-deficient iPSC-precursors did not improve, but further suppressed the reprogramming. Our data therefore point to distinct modes of mitochondrial ROS action during the early versus mid and late stages of reprogramming. The data further substantiate the paradigm that balanced levels of oxidative phosphorylation have to be maintained on the route to pluripotency.

## 1. Introduction

Embryonic stem cells (ESCs) are derived from the inner cell mass (ICM) of a blastocyst stage during embryo development and have a unique capacity to differentiate into any somatic cell type of an adult organism [1]. Cells with similar characteristics can also be derived via the reprogramming of somatic cells with ectopically-expressed transcription factors Oct4, Klf4, Sox2, and c-Myc, therefore designated as induced pluripotent stem cells, or iPSCs [2]. To date, iPSC technology is extensively used for disease modelling and therapeutic drug development. Another very promising iPSC application resides in regenerative medicine and tissue replacement. However, these applications have several challenges so far, mainly because of some differences in quality and resemblances of iPSCs to ESCs [3,4,5,6,7,8,9]. Importantly, recent studies showed that procedures of iPSCs generation and subsequent cultivation in vitro predispose them to increased rates of genomic abnormalities and mutations [6,10,11]. One of the causes leading to genome instability and molecular lesions is high levels of reactive oxygen species (ROS) generated by abnormal functions of mitochondria and dysregulation of antioxidant defense systems [12,13]. In this regard, it is of high priority to investigate metabolic and redox mechanisms underlying cell reprogramming for the pluripotent state.

Vital physiological functions of moderate sublethal ROS levels or oxidative eustress in various cell and developmental processes, including cell reprogramming during pluripotency, are well established. During reprogramming, cells undergo major changes in redirecting metabolic fluxes, mitochondrial network dynamics and function, and regulating redox-related cell signaling network [14,15,16,17,18]. During this process, a switch from dominant oxidative phosphorylation (OxPhos) of somatic cells to glycolytic metabolism of pluripotent cells, which is associated with extensive mitochondria reorganization and ROS regulation, occurs [17]. Cell reprogramming to pluripotency requires tight regulation of ROS levels and metabolic fluxes [16]. It has been shown that optimal ROS signaling induced by the nicotinamide adenine dinucleotide phosphate (NADPH) oxidase 1–4 (Nox1–4) complex is required during the early stages (first 7 days) of cell reprogramming [19]. Importantly, it was shown that low oxygen tension or hypoxic conditions, which are normally presented in embryonic epiblasts, are critically important for the generation and culturing of ESCs and iPSCs, given that they reduce the accumulation of genetic lesions [12,20].

Mitochondria are a cell organelle that participates in a redox regulation together with their major functions: energy generation and regulation of metabolic pathways [21]. In mitochondria, energy or adenosine triphosphate (ATP) generation is mediated through oxidative phosphorylation, which involves the permanent activity of the respiratory electron transport chain (ETC). ETC consists of 5 multiprotein complexes that are localized in the mitochondrial inner membrane, which mediates electron acceptance and transfer from NADH and FADH to oxygen and is accompanied by proton transport across the inner membrane and results in a proton gradient that is essential for ATP generation by ATP-synthase. As a byproduct of the oxidative phosphorylation, some electrons escape from ETC, which results in superoxide anion (SOA, O_2_^−^) generation. The main contributor to ROS generation by ETC is the complex I (CI), which is represented by the 45-subunit NADH-ubiquinone oxidoreductase [22,23,24]. SOA is deposited in the mitochondrial matrix [24,25,26,27,28,29], then it is transported from mitochondria to cytosol by voltage-dependent anion channels [30]. A part of SOA is also released by the ETC Complex III (CIII) into both the intermembrane and matrix spaces. SOA is a major and highly-reactive form of ROS derived from mitochondria, which is dismutated to hydrogen peroxide (H_2_O_2_) spontaneously or by superoxide dismutases 1–3. Hydrogen peroxide is a dominant form of ROS in the cell, which has vital physiological functions in modifying various signaling proteins. The generated ROS levels vary depending on the status of the ETC activity. The regulation of ROS production by various components of ETC in different cellular contexts can induce specific physiological signaling events [14,31,32,33]. In a *Drosophila* model, the genetic silencing of the Ndufs1 or Ndufb10 subunits of CI leads to ROS generation, thereby mediating different retrograde signaling that regulates hematopoiesis, cell cycle, and cellular defense response [14,34,35,36,37]. However, the function of CI subunits in other mammalian cell types or during the process of cell reprogramming has not been studied.

Here, we have investigated the roles of mitochondrial ROS during the cell reprogramming process. The sources and level of ROS are critically important for the biological functions of secondary messengers. We show that knockdown of Ndufs1 or Ndufb10 subunits of CI or treatment with its inhibitor, rotenone, induce strong ROS generation, which results in a suppression of the reprogramming process. Our data suggest that different levels of ROS are required at different stages of cell reprogramming. The highest levels of ROS, reached by day 3 of reprogramming, have a negative effect on the progression of the process. Attenuating ROS levels at this stage ameliorates, while doing so during intermediate or late phases suppresses reprogramming to the pluripotent state, thereby suggesting the dual mode of ROS functioning during this process. The data also indicate that ETC CI activity during reprogramming is critically important to establish pluripotency.

## 2. Results

### 2.1. shRNA-Mediated Knockdown of Ndufs1 or Ndufb10 Leads to an Increased ROS Production in Somatic Cells and ESCs

Short hairpin RNA (shRNA)-encoding constructs directed to Ndufs1 and Ndufb10 transcripts were generated to investigate the function of CI during the process of reprogramming somatic cells into a pluripotent state. Three lentivirus constructs for each of the mRNAs were tested to suppress these mRNA in mouse embryonic stem cells (ESCs) and somatic cells (STO cell line). The Ndufs1 shRNAs, sh927 and sh2350, strongly suppressed Ndufs1 mRNA expression in STO cells and ESCs, while sh330 did so to a limited extent (Figure S1A,B). Ndufb10-specific shRNA constructs (sh400, sh511, and sh570) also completely suppressed the expression of the target mRNA in STO cells and ESCs (Figure S1C and data not shown). We also noticed that Ndufb10 was expressed in ESCs at a significantly lower level (3–4 times), compared to that in primary MEFs (data not shown, [38]).

**Figure 1:**
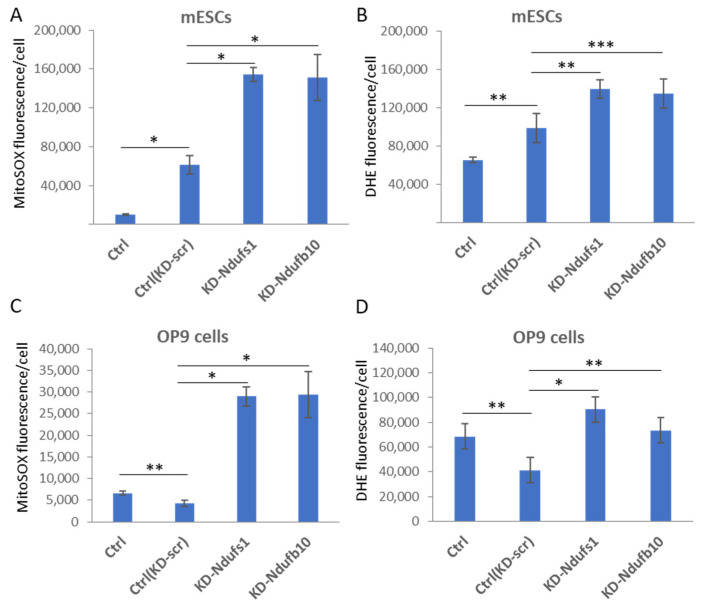
Inactivation of Complex I (CI) of mitochondrial ETC leads to an increased ROS production in pluripotent and somatic cells. shRNA-mediated knockdown (KD) of Ndufs1 or Ndufb10 mRNA elevates mitochondrial superoxide anion (**A**,**C**) and total ROS (**B**,**D**) production in mouse ESCs (**A**,**B**) and the mouse mesenchymal stem cell line OP9 (**C**,**D**). Control shRNA (KD-Scr) decreases ROS levels in OP9 cells (**C**,**D**), whereas it augments ROS levels in ESCs (**A**,**B**). *Y*-axis represents mean fluorescence of MitoSOX-red (**A**,**C**) and DHE staining (**B**,**D**) per cell ± standard deviations (±SD), *n* = 3, * *p* < 0.001, ** *p* < 0.01, *** *p* < 0.05.

We next found that Ndufs1 or Ndufb10 knockdown in both somatic and ESCs led to the generation of intracellular superoxide anion and total ROS. ESCs were transduced with lentiviruses carrying Ndufs1- or Ndufb10-specific shRNA, then cultured in puromycin-containing selective medium for 4 days. The levels of mitochondrial superoxide anion and total ROS in cells were measured by flow cytometry using MitoSOX and DHE staining, correspondingly. Compared to scrambled (scr) shRNA control, inactivation of Ndufs1 or Ndufb10 caused 2,5- and 7-fold increase of SOA production in ESCs and OP9 cells, respectively (Figure 1A,C). In addition, total ROS levels were also significantly elevated upon Ndufs1 or Ndufb10 inactivation in ESCs (1.4-fold) and OP9 cells (2-fold), when compared with scrambled control (Figure 1B,D). Scramble shRNA by itself caused a significant increase of SOA and total ROS levels in ESCs, however, significantly reduced ROS levels in OP9 cells (Figure 1). Notably, the increase of total ROS production was less pronounced than the increase in mitochondrial SOA, suggesting specific production of mitochondrial ROS upon CI malfunction. These data indicate that inactivation of these two CI dehydrogenase subunits causes a strong increase in the level of mitochondrial superoxide anion in both somatic and pluripotent stem cells.

### 2.2. Knockdown of Ndufs1 or Ndufb10 Significantly Reduces the Efficiency of Cell Reprogramming to Pluripotent State

**Figure 2:**
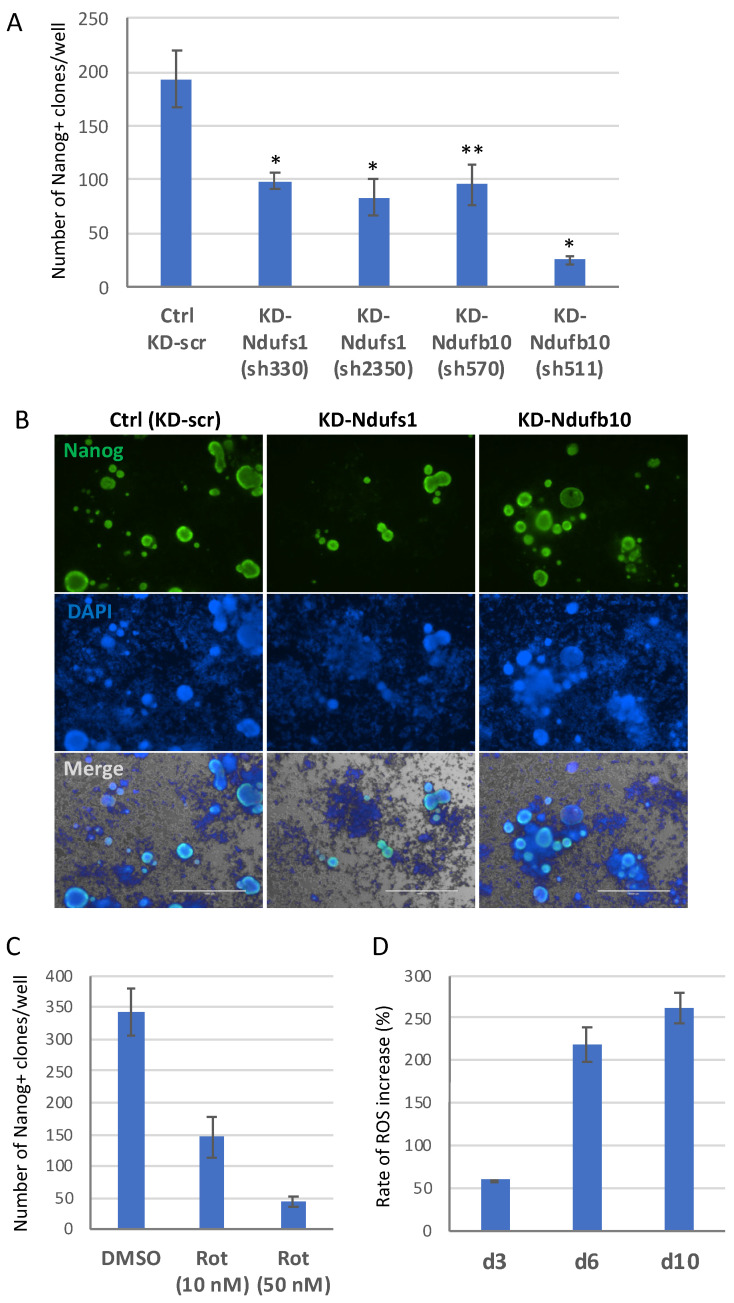
Functions of Ndufs1 or Ndufb10 are important for efficient reprogramming into the pluripotent state. (**A**) Knockdown (KD) of Ndufs1 or Ndufb10 during the whole reprogramming process greatly reduces the efficiency of reprogramming with OSKM. Two independent shRNA constructs targeting Ndufs1 or Ndufb10 mRNAs strongly suppress the generation of Nanog-positive iPSCs clones from MEFs, compared with the control (KD-scr). The y-axes indicate the number of Nanog-positive clones on day 14 of reprogramming ±SD, *n* = 3, * *p* < 0.005, ** *p* < 0.01. (**B**) Images of day 14 iPSC clones generated with Ndufs1 or Ndufb10 mRNA being knocked down, as revealed by anti-Nanog (green) immunofluorescence staining (merge images contain Nanog, DAPI staining (blue) and phase-contrast image, scale bar—1000 μm). (**C**) CI inhibition by rotenone (Rot) at 10 nM and 50 nM leads to a strong suppression of the reprogramming compared to the DMSO control; results are shown as the numbers of Nanog-positive clones ±SD, *n* = 3, *p* < 0.001. (**D**) Treatment with 10 nM rotenone during the reprogramming caused a strong increase of ROS level in iPSC precursors, shown by DHE staining. The *y*-axis represents means of ROS elevation rate (%) by rotenone at day 3, 6, and 10 of the reprogramming, compared to DMSO-treated cells on the corresponding day of reprogramming (±SD, *n* = 2, *p* < 0.005).

To investigate effects of mitochondrial ROS and impaired mitochondrial function on the process of MEF reprogramming into iPSCs, we used the same Ndufs1- and Ndufb10-directed shRNAs constructs (Figure S1). These shRNAs constructs were used to knockdown corresponding mRNA throughout the reprogramming induced by the OKSM reprogramming factors. Knockdown of Ndufs1 or Ndufb10 by the two different shRNAs during the entire reprogramming period caused a significant (2-times) decrease in the efficiency of iPSC generation (Figure 2A). The developed KD-Ndufs1 and KD-Ndufb10 iPSC clones were morphologically similar to those obtained using scramble shRNA control and expressed pluripotency markers Nanog and SSEA1 (Figure 2B, Figures S2 and S4). However, it was noticeable that by day 14 of reprogramming, KD-Ndufs1 iPSC clones were smaller while KD-Ndufb10 were larger in size than KD-Scr controls. Importantly, almost all KD-Ndufs1 clones after the second passage tended to spontaneously differentiate, as evidenced by the loss of Nanog and SSEA1 expression (Figures S2 and S3). The same was noticed for KD-Ndufb10 clones (Figure S4). These results suggest that ETC CI activity is beneficial for reprogramming to the pluripotent state, while it becomes essential for the maintenance of this state in resulted iPSCs. In addition, we noticed that upon inactivation of ETC CI during reprogramming, iPSCs colonies developed earlier than control ones. This was especially true for KD-Ndufb10, as evidenced by a significant increase of SSEA1^+^ iPSC-precursors by day 10 of the reprogramming (Figure S5). All together, these data indicate that ETC CI is critically important for the establishment of pluripotent state.

In addition, inactivation of ETC C1 with 10 nM and 50 nM of the specific low-molecular-weight inhibitor rotenone during the entire process of cell reprogramming also resulted in an approximately 2- and 7-fold reduction of iPSC clone numbers, respectively (Figure 2C). Of note, rotenone at the concentration of 10 nM did not affect cell viability, whereas 50 nM it caused about a 2-fold increase of apoptosis (Figure S6A,B). Importantly, we confirmed that 10 nM rotenone treatment induced robust elevation of ROS levels in iPSC-precursors during all stages of the cell reprogramming process (Figure 2D and Figure S7), as well as that it sufficiently suppressed the mitochondrial membrane potential, as measured by a JC ratio assay (Figure S6C). These results indicate that the function of mitochondrial ETC CI is important for cellular reprogramming into a pluripotent state and that elevated mitochondrial ROS are responsible for the suppression of the cell reprogramming process.

### 2.3. ETC CI Suppression Has Stage-Specific Effects on Reprogramming

**Figure 3:**
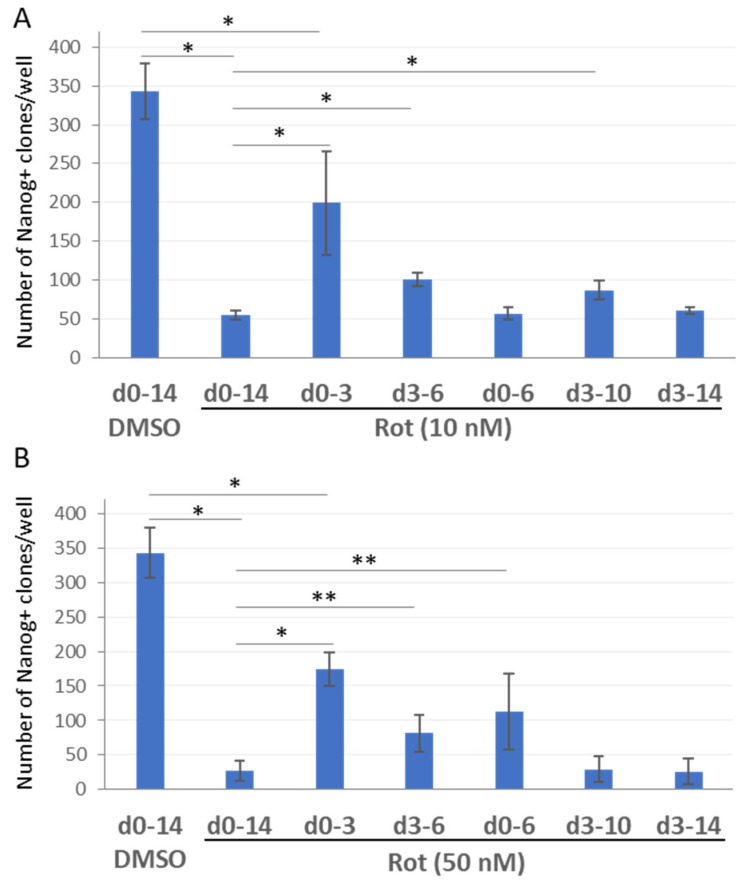
ETC CI suppression by rotenone shows stage-specific effects on reprogramming outcomes. (**A**) Rotenone treatment at specified concentrations during the first 3 days of reprogramming exerts a significantly lesser effect on iPSC clone number than the treatment during the first 6 or 10 days, or during 3–10 or 3–14 days of the reprogramming process. (**B**) Rotenone treatment (50 nM) during the first 3 and 6 days causes a significant increase in cellular reprogramming, when compared with its treatment during the first 10 days, or during 3–10 or 3–14 days of the reprogramming process. In both charts, the *y*-axis represents the mean number of the Nanog-positive clones by day 14 of reprogramming ±SD, *n* = 3, * *p* < 0.001, ** *p* < 0.01. The *x*-axis indicates the time intervals (in days) of rotenone treatment during reprogramming process.

To investigate the roles of mitochondrial ETC CI during different stages of the reprogramming, we performed rotenone treatments during variable time intervals of the process. CI inactivation with rotenone (10 or 50 nM) during the first 3 days of the reprogramming had a significantly lesser negative effect on iPSC generation, compared to the full term (day 0–14) or time-limited exposure (days 3–6, 0–6, 3–10, 3–14) to this chemical (Figure 3). It is likely that loss of CI function upon rotenone treatment during mid and late reprogramming phases results in an elevated mitochondrial ROS production, which has a negative effect on the progression towards a mature iPSC state. As opposed to that, ROS increase during the initial phase barely affects the reprogramming outcomes. This conclusion agrees with large-scale rearrangements of cellular metabolic flows, including the rearrangement and fission of the mitochondrial network, occurring at the initial stage of reprogramming [38,39]. It is also likely that the reduced sensitivity of the reprogramming to rotenone-mediated inhibition of ETC CI during the first 3 days is because the ROS (or their downstream effectors) are already at levels close to saturation. The experiments reported below support this viewpoint.

### 2.4. The Dual Function of ROS during the Reprogramming

**Figure 4:**
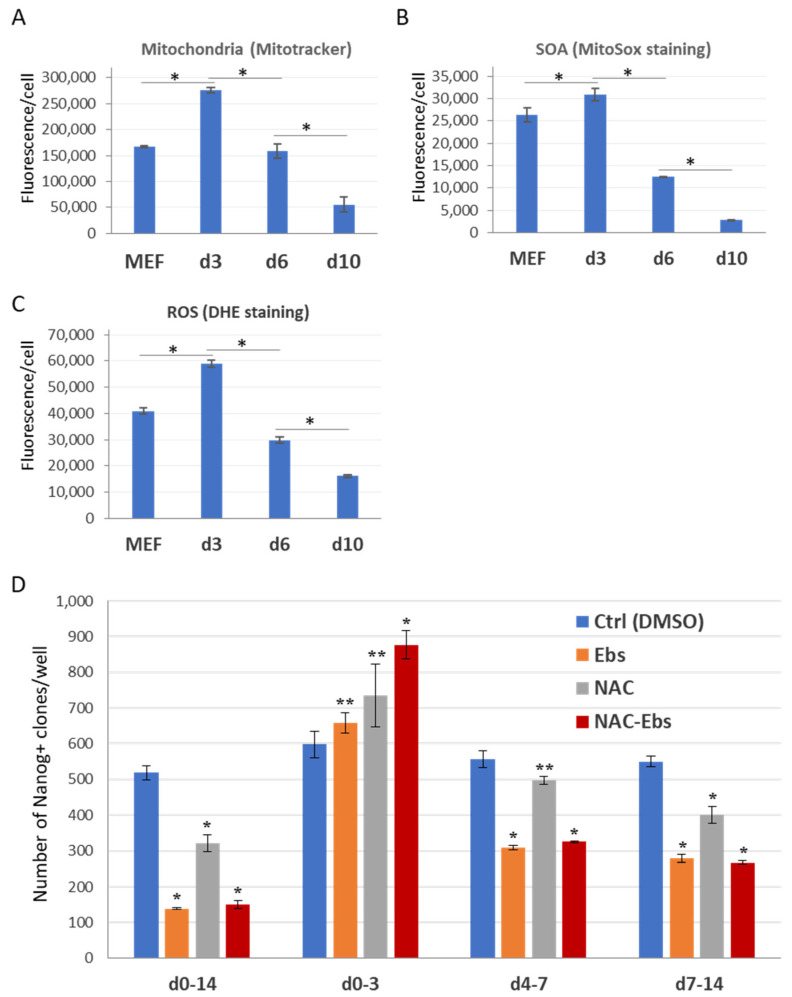
ROS exerts opposite effects during early (day 0–3) vs. later phases (day 4–14) of reprogramming. Transient increase of mitochondria numbers (**A**), SOA (**B**), and ROS (**C**) on day 3, followed by their dramatic decrease by day 10 of reprogramming. The parameters were measured by Mitotracker, MitoSox, and DHE staining, respectively. The charts show mean fluorescence per cell ±SD, *n* = 3, * *p* < 0.001. (**D**) The ROS scavenging with antioxidants between day 0 and 3 of reprogramming increases, whereas after day 4 it reduces the efficiency of iPSC generation. The chart shows mean of Nanog-positive iPSCs clone numbers per well ±SD, *n* = 3, * *p* < 0.001, ** *p* < 0.05.

Next, we measured mitochondrial dynamics and levels of ROS generation on day 3, 6, and 10 of the reprogramming. Interestingly, mitochondria content dropped about 8 times from day 3 to day 6 of the reprogramming, as shown by the Mitotracker-red FACS assay (Figure 4A and Figure S8). Mitochondria amounts further decreased 23% from day 6 to 10. In agreement with this, mitochondrial SOA and total ROS levels are reduced 2.5- and 2-fold in iPSC-precursors during the reprogramming progress from day 3 to 6, correspondingly (Figure 4B,C and Figure S8). From day 6 to 10, these ROS levels further decreased 4.4- and 1.9-times, respectively (Figure 4B,C). These data indicate that during the cell reprogramming, the mitochondria network undergo major changes: its amounts reaches the highest by day 3 of the process, then gradually declines during subsequent stages, perhaps through the processes of mitochondria fission and mitophagy [40]. These changes are accompanied by a major decrease in SOA generation in immature and mature iPSCs. Thus, iPSCs precursors inherently possess high numbers of mitochondria and elevated ROS levels by day 3 of reprogramming, followed by their gradual decline upon further advancement to pluripotency state. It also seems that early iPSC precursors resist oxidative stress better than their more mature counterparts (see below).

To find out whether mitochondrial ROS mediates the effects of Ndufs1 knockdown and rotenone treatment on cellular reprogramming, we made use of two known antioxidants: N-acetyl-L-cysteine (NAC) and ebselen (Ebs). First, we have evaluated if continuous treatment with these antioxidants affects cell reprogramming. Optimal working concentrations of the antioxidants were determined by their effect on DHE intensity in ESCs and MEFs (Figure S9 and data not shown). The treatment with Ebs (5 μM) or NAC (2 mM) during the entire process of reprogramming leads to a 3-fold (Ebs or Ebs + Nac) or 1.5-fold (NAC) reduction of iPSC clone numbers (Figure 4D). This result indicates that certain ROS levels are important for cell reprogramming, which is consistent with previously published data [19]. To discriminate the roles of ROS during the onset (1–3 day), intermediate (4–7 day), and final (7–14 day) stages of reprogramming, the antioxidants were applied during the indicated time intervals. Interestingly, the ROS scavenging at an early stage, i.e. during days 1–3 of cell reprogramming, significantly enhanced (by 10–47%) its outcome (Figure 4D). On the contrary, ROS scavenging by antioxidants during the periods of day 4–7 or 7–14, reduced the efficiency of the cell reprogramming 1.4- or 2-times, respectively (Figure 4D). This observation suggests that optimal levels of ROS are important at all stages of the reprogramming process. Therefore, there are at least two modes of ROS action during MEF reprogramming to pluripotent state. During the early phase of this process, initially high levels of ROS in MEFs further increase, which has a negative effect on reprogramming success. However, during intermediate and late phases, reduced ROS levels positively regulate the cell reprogramming process, while iPSC-precursors demonstrate a reduction of mitochondria and oxidative phosphorylation, accompanied by a dramatic decline of ROS levels. However, even the lowered mitochondrial ROS levels at these stages of reprogramming are critical for reprogramming.

### 2.5. ROS and ETC CI Functions Are Essential in the Process of Cellular Reprogramming

**Figure 5:**
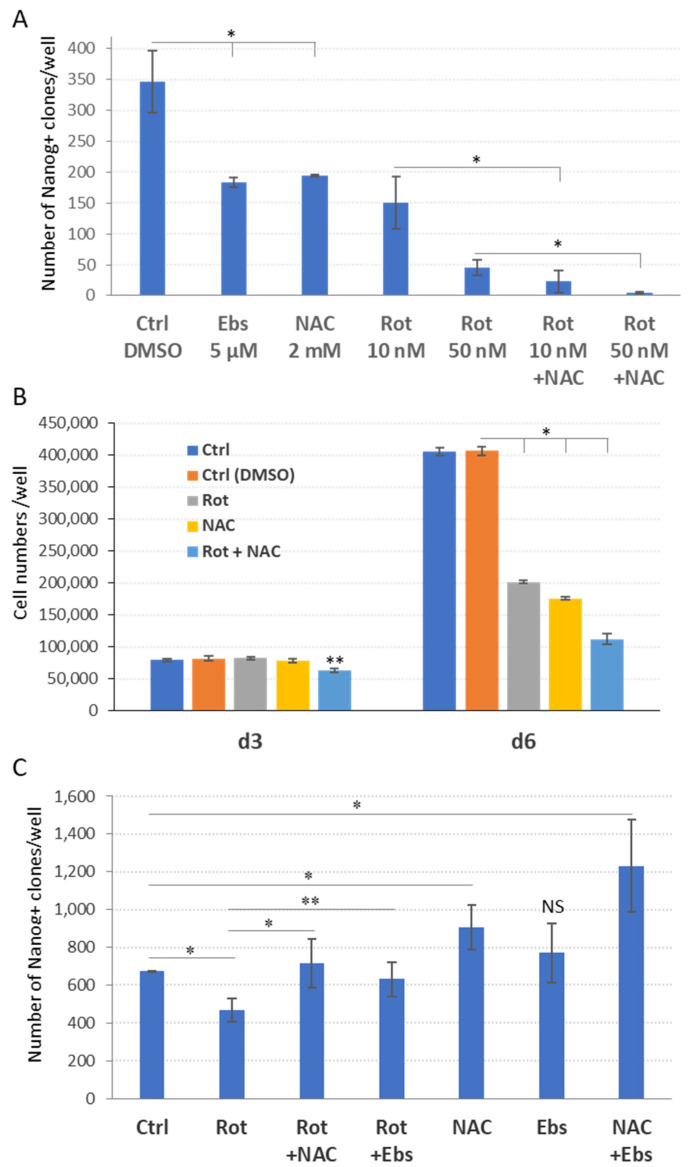
ROS and OxPhos interaction during cell reprogramming. (**A**) ROS scavenging exacerbates the ETC CI deficiency-induced suppression of iPSC formation in the context of full-term reprogramming. NAC or Ebs and rotenone (Rot) were applied as indicated during the whole reprogramming period, i.e., for d0–14 (±SD, *n* = 3, * *p* < 0.005). NAC (2 mM) exacerbates the repressive effect of rotenone (±SD, *n* = 3, * *p* < 0.005). (**B**) Total cell counts by FACS revealed that treatment with NAC, Rot, or both substances cause significant reduction of cell numbers on day 6 (±SD, *n* = 3, * *p* < 0.005), but not on day 3 of the reprogramming course (*n* = 3, ** *p* < 0.05). (**C**) ROS scavenging during the early phase of reprogramming attenuates the effect of ETC CI inhibition on iPSC formation. Rotenone (10 nM), NAC (2 mM), and Ebs (5 μM) were added for the period of day 0–3 as denoted. Rotenone (10 nM) treatment decreases the reprogramming efficiency, compared to the control (Ctrl, DMSO, ±SD, *n* = 3, * *p* < 0.01). Antioxidant treatment attenuates the repressive effect of rotenone (±SD, *n* = 3, ** *p* < 0.02). NAC or NAC + Ebs (*n* = 3, * *p* < 0.01), but not Ebs alone, promotes the reprogramming efficiency compared to the control (Ctrl, DMSO). The *y*-axis indicates the number of Nanog^+^ iPSCs colonies by day 14 of the reprogramming.

Next, we investigated whether the ROS scavenging by antioxidants could reverse the suppressive effect of rotenone on cell reprogramming process. Consistent with previous results (Figure 3 and Figure 4D), rotenone and NAC inhibited reprogramming when applied separately (Figure 5A). Surprisingly, however, the treatment with NAC throughout the entire process of reprogramming did not attenuate the suppressive action of rotenone but rather exacerbated it, resulted in further decrease (6–11 times) of iPSC colony numbers (Figure 5A). The obtained results indicate a synergistic effect of antioxidants and impaired CI on reprogramming, suggesting that unaccounted factors other than ROS are involved in the reprogramming process. Of note, the reduction of reprogramming via full-term treatment with either antioxidants or rotenone (or both) correlates with a strong reduction of proliferation of iPSC-precursors on day 6 of the process (Figure 5B). This reduction is unlikely to result from increased apoptosis or necrotic cell death (Figure S6A,B and our unpublished data). In addition, to robustly attenuate the mitochondrial function, we used a protonophore carbonyl cyanide m-chlorophenylhydrazone (CCCP) which depolarizes the inner mitochondrial membrane to uncouple oxidative phosphorylation from the ETC. As expected, CCCP treatment during days 4–14 or during the entire reprogramming results in a strong suppression of the process (Figure S10). Thus, these data indicate that both optimal ROS levels and full capacity oxidative phosphorylation are critical for reprogramming to pluripotency.

As opposed to the above result, ROS scavenging during the early phase of reprogramming (days 0–3) essentially attenuates the inhibitory effect of ETC CI inhibition on iPSC formation (Figure 5C). Consistent with previous results (Figure 4D), antioxidants applied during this period alone also ameliorated reprogramming (Figure 5C). These data additionally support the conclusion that excessive ROS is detrimental for reprogramming. 

**Figure 6:**
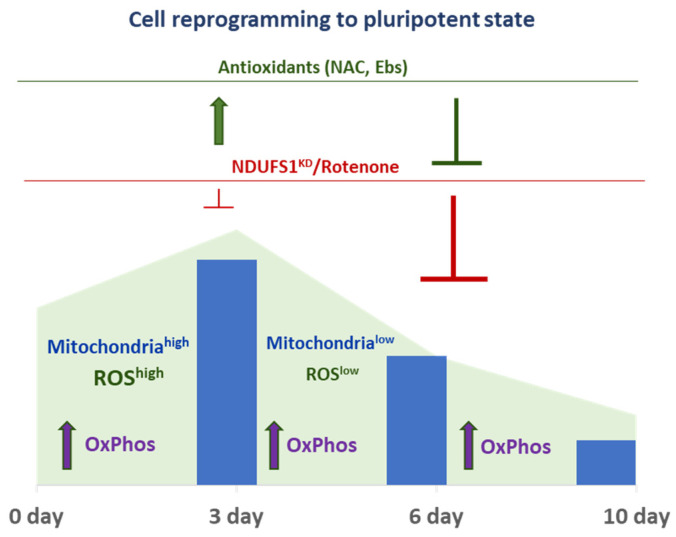
Summary diagram showing dynamics of mitochondria content and ROS generation during the course of reprogramming of MEFs to iPSCs. Inhibiting CI of ETC via Ndufs1/Ndufb10 knockdown or treatment with rotenone are well tolerated during the first 3 days, however, strongly suppresse cell reprogramming during intermediate and late stages of this process. ROS scavenging by antioxidants ameliorates cell reprograming during the first 3 days, however, it suppresses the process when applied at later stages.

These obtained data also suggest that metabolic changes, such as the decrease of oxidative phosphorylation and ROS, which occur during intermediate and late stages, are tightly maintained and required for efficient cell reprogramming (Figure 6). In summary, in iPSC-precursors during the first 3 days of reprogramming, major changes of mitochondrial content and function associated with high levels of mitochondrial ROS production occur (Figure 6). By this stage, both deficiency of oxidative phosphorylation and ROS increase, induced by CI function loss, are well tolerated by iPSC precursors. However, later (after day 3) mitochondria and ROS levels are majorly reduced, and these optimal levels of ROS and oxidative phosphorylation are required and sufficient for reprogramming iPSC precursors to term.

## 3. Discussion

The reprogramming of somatic cells to iPSCs includes the transition from OxPhos to glycolysis metabolism, which is accompanied by the upregulation of glycolytic genes and the downregulation of OxPhos genes. The activation of pyruvate dehydrogenase kinase 1 (PDK1), which facilitates the metabolic transition to glycolysis, combined with single reprogramming factor Oct4, are sufficient for achieving the pluripotency state [17], thereby supporting the idea that the stimulation of the glycolytic flux promotes reprogramming [41]. Beside dramatic changes of mitochondria energy metabolism during the reprogramming, processes associated with the structural, morphological, and functional conversion of mitochondria into an immature state makes this organelle an important signaling and metabolic hub that participates in pluripotency establishment.

The present study shows that the function of mitochondrial ETC CI is important for the cell reprogramming to the pluripotent state. Continuous suppression of CI function during the entire process of reprogramming leads to a suppression of iPSC generation. Mitochondria content dramatically changes in iPSC-precursors, reaching its peak on day 3 and subsequently dramatically declining by day 6, thereby suggesting a significant reorganization of the mitochondrial network during the transition from somatic to pluripotent states. This conclusion is consistent with earlier studies which have shown that mitophagy occurs during cell reprogramming [40] and OxPhos capacity reaches a peak by day 2–3 of reprogramming [39]. Importantly, mitochondrial SOA and total ROS also peak in iPSC-precursors by day 3, subsequently declining by day 6 and 10, which correlates with observed mitochondria dynamics during these reprogramming stages (Figure 6). Moreover, additional ROS generated by an inactivation of ETC CI during the first 3 days are largely tolerated and permissive for the reprogramming, while inactivation of this complex at later stages causes a strong suppression of the reprogramming. These results suggest that in addition to Nox (1–4)-generated ROS [19], mitochondria normally produce high levels of ROS during the early stages of reprogramming, which largely contributes to the success of the process [39].

Second, due to the significantly reduced number of mitochondria during the mid- and late stages, the impact of mitochondrial ROS during these stages of reprogramming is not as clear. Inactivation of CI during these stages, which is associated with increased ROS generation, results in a significant reduction of reprogramming efficiency. However, it is plausible that this reduction is also caused by the metabolic switch from OxPhos to glycolysis or by other metabolic changes associated with CI deficiency [17]. Ndufs1 deficiency also caused a decrease of OxPhos, as evidenced by the reduced mitochondrial membrane potential (MMP) and mitochondrial content in cardiomyocytes [42] and in ESCs (our unpublished data). On average, CI contributes about 40% of the proton gradient force required for mitochondrial ATP synthesis [43]. However, our data indicate that knockout of Ndufs1 in mouse ESCs increases the total ATP production (our unpublished data). It was also shown that inhibiting OxPhos by the CCCP uncoupler resulted in a 30% and 40% ATP drop in ESCs and their differentiation progeny, correspondingly [44]. This result suggests that the impact of CI on the total ATP production is cell-type dependent. It is possible that the CI-mediated influence of ATP generation at various stages of cell reprogramming may take place, and the proposal deserves further investigation. On the other hand, ROS scavenging during the mid and late stages significantly suppresses iPSC generation. This result suggests an important function of mitochondria during these stages of reprogramming, i.e. when activation of glycolysis takes place [38]. It is also possible that non-mitochondrial ROS are involved in the vital regulation of the intermediate reprogramming stage. 

The importance of optimal OxPhos and ROS levels during intermediate and late stages is supported by the data that simultaneous treatment with rotenone and antioxidants demonstrates a synergistic effect toward the robust suppression of reprogramming. Thus, both optimal ROS levels and OxPhos during the mid and late stages are required for efficient iPSC generation. Importantly, our data show that sufficiently high levels of mitochondrial ROS are required at the onset of reprogramming, however, this level is also somehow detrimental to the process (Figure 6). The removal of the ROS excess during the first 3 days mainly improves the efficiency of the pluripotency reprogramming. Oxidative burst occurring during an early stage of the reprogramming has been previously observed [39]. Estrogen-related nuclear receptor gamma (ERRγ) together with PPAR-γ co-activators, PGC-1α and PGC-1β, are the main factors regulating this metabolic burst in iPSC-precursors during day 3 of the reprogramming. The removal of ERRγ leads to an almost complete block in cell reprogramming, and this ERRγ function is required during the first 3 days of the process before establishing pluripotency [39]. Kida et al. showed that in Sca1^−^;CD34^−^ double negative *bona fide* iPSC-precursors, ERRγ and PGC1β, as well as a number of OxPhos mitochondrial genes, including Complexes I-V ETC, are upregulated [39]. Two other factors, Zic3 and Esrrb, are also involved in the repression or activation of oxidative phosphorylation during the reprogramming process [45]. A quantitative proteomic analysis showed highly coordinated changes in the expression and stoichiometry of ETC complexes during the first 3 days and intermediate stages of reprogramming [38]. The decreased expression of ETC complexes I and IV, as well as increased expression of complex II, III, and V subunits, which occur during the early stage, might also contribute to increased ROS generation, associated with a change in oxidative phosphorylation [46,47]. Thus, even with the requirements of this early oxidative burst and excess of ROS for the success of reprogramming, sufficient downregulation of ROS at this stage significantly enhanced the process. 

In sum, our present study thus substantiates an importance of mitochondria function and mitochondria-generated ROS during the process of reprogramming to iPSCs, which might potentially allow to improve quality of iPSCs, thereby promoting the introduction of these promising cells into regenerative medicine.

## 4. Materials and Methods

### 4.1. Cell Culture

Murine embryonic fibroblasts (MEFs) were isolated from 13,5–14,5 dpc embryos of C57BL/6 mice. MEFs, STO, OP9, and HEK293T cells were grown in medium consisting of DMEM media, 10% FBS, 100 U/mL penicillin, 100 mg/mL streptomycin, and 2 mM L-Glutamine. Most cell culture media, supplements, and other chemicals in this study were from Thermo Fisher Scientific, Waltham, MA, USA, if not specified. Mouse ESCs and iPSCs were cultured in mouse embryonic stem (MES) medium: knockout DMEM media, 15% FBS (Biosera, Nuaille, France), 100 U/mL penicillin, 100 mg/mL streptomycin, 2 mM L-Glutamine, 1μM non-essential amino acids NEAA, 50 μM β-mercaptoethanol (β-MET, Sigma-Aldrich, St. Louis, MO, USA), and in-house produced leukemia inhibitor factor LIF (1:5000). N2B27 2i media containing standard N2B27 medium was used: 1/2 Neurobasal, 1/2 DMEM/F12, 100 U/mL penicillin, 100 mg/mL streptomycin, 2 mM L-Glutamine, 25 μM β-MET (Sigma-Aldrich, St. Louis, MO, USA) supplemented with N2 (dilution 1:100) and B27 (dilution 1:50), 3 μM CHIR-99021, 1 μM PD-0325901 (Axon Medchem, Groningen, The Netherlands), recombinant hLIF (5 ng/mL), and 3 μg/mL Dox at 37 °C in a standard CO_2_ incubator.

### 4.2. Short Interfering RNA Constructs

Short interfering RNA (shRNA) constructs for Ndufs1 and Ndufb10 genes were developed with the use of pLKO.1 vector (Addgene #10878). PCR-amplified DNA fragments were selected by (addgene/plko.protokol) and cloned into the vector by the AgeI and EcoRI sites. DNA fragments were used—from Ndufs1 cDNA: 330–352 bp AAGGTTGTCTGTTGCTGGAAA (shRNA 330); 927–949 bp AACTGGAGAGGTAATGAGGAT (shRNA 927); 2350–2372 bp AATGGACATTGCTGTAATCCC (shRNA 2350); from Ndufb10 cDNA: 378–400 bp AAAGTTGACCAGGAGATCATG (shRNA 400); 489–511 bp AACTCTTCATTCCACTGCTAA (shRNA 511); 548–570 bp AAAGTGTCTGGCAAAGCAGAA (shRNA 570). The fragments were ligated by DNA ligase (C-E320 T4, SibEnzyme). The cloned constructs were sequenced with primers for U6-D1 (5′GTAAACACAAAAGATATTAGTACAA3′).

### 4.3. Lentiviral Particles Preparation

Lentivirus packaging was performed as described before. HEK293T were co-transfected with envelope (pMD2G, 4 μg) and packaging (psPAX2, 8 μg) plasmids and various target plasmids (11 μg for each): pLKO-Ndufs1-shRNA-300, -927, -2350; pLKO-Ndufb10-shRNA-400, -511, -570, pLKO-scramble-shRNA; or M2rtTA or pHAGE2-OKSM plasmid, carrying reprogramming factors (Oct4, Klf4, Sox2, cMyc) (Sommer et al., 2009 [48]) with the use of 40 µg polyethylenimine (PEI) transfection reagent (PEI MAX^®^ MW 40000, Polysciences, Inc., Warrington, PA, USA). After the mixture was incubated for 15 min, it was added to the cells and incubated for 18 h in antibiotic-free MEF media. Virus particles were collected by centrifugation at 47,000 rpm at 4 °C for 2 h from the media supernatants, were dissolved in 150 µL of OptiMEM medium, and then kept at −80 °C [49,50,51]. The titer of viral particles was determined by puromycin selection and constructed in a range of 1–2 × 10^7^ TU/mL.

### 4.4. Transduction with Lentiviruses

ESCs (E14 Tg2a), immortalized MEFs (STO), or mouse mesenchymal stromal cells (OP9) in the logarithmic growth phase were transduced by an optimal number of lentiviruses in OptiMEM media for 18 h (200 µL/well of 24 w/p). Cells were grown in complete growth medium, with 2–5 µg/mL puromycin appropriately used for each type of cell.

### 4.5. Reprogramming of MEFs to iPSCs

Cell reprogramming was performed as described [52]. MEFs were grown in standard MEF media in a CO_2_-incubator: 5% CO_2_ at 37 °C. Cells were seeded (3 × 10^4^ cells per well) on 0.1% gelatin-coated 24-well plate in the MEF medium. Next day media was replaced with 200 μL of Opti-MEM media (Gibco) containing mixture of lentiviruses (MOI = 2–5 for each): M2rtTA + pHAGE2-OKSM [48] and different shRNA-pLKO. Cells were incubated with the virus mixtures for 3–4 h, then 200 μL of Opti-MEM were added and incubation was continued overnight. Next day media was changed to MEF media containing 3 μg/mL Dox, and supplemented with chemicals corresponding to the particular experiment: 10 or 50 nM Rotenone (Rot, Abcam, Cambridge, UK), 2 mM N-acetyl-cysteine (NAC, #A7250, Sigma-Aldrich, St. Louis, MO, USA), 5 μM Ebselen (#5245/10, Tocris Bioscience, Bristol, UK), and a protonophore carbonyl cyanide m-chlorophenylhydrazone (CCCP, ab141229, Abcam, Cambridge, UK). Media was changed every day, and on the 3rd day, cells were trypsinized and seeded onto 3 wells of 12-well plates that were pre-plated mitomycin C-treated MEFs. Cells cultured in N2B27-2i media had the media changed every other day. On day 14–15, developed iPSC clones were fixed and proceeded to immunostaining or harvesting. For characterization, six iPSC clones of each knockdown line were picked up and grown in MES media. For ROS and mitochondria measurements, the reprogramming was performed in 12-well plates during the first 6 days without replacement to feeder cells, but with N2B27-2i media replacement on the 3rd day, then on day 6, and cells were trypsinized and transferred to 3 wells of 12-well plates containing pre-plated mitomycin C-treated MEFs and cultivated until day 10.

### 4.6. Fluorescence-Activated Cell Sorting (FACS) Measurement of ROS and Mitochondria

Cells in the logarithmic growth phase or at a corresponding reprogramming stage in a 12- or 24-well plate were rinsed with PBS and incubated with 150 µL of 5 µM MitoSOX™ Red (Thermo Fisher Scientific, Waltham, MA, USA) or 1 µM of dihydroethidium solution (Dihydroethidium, DHE, #ab145360, Abcam, Cambridge, UK) or Mitotraker (Thermo Fisher Scientific, Waltham, MA, USA) in MEM medium for 30 min at 37 °C in a CO_2_ incubator. Cells were harvested by standard trypsinization with a 0.05% trypsin-EDTA solution. After centrifugation, cells were resuspended in 150 μL of PBS and analyzed on a CytoFLEX FACS flow cytometer (Beckman Coulter, Pasadena, CA, USA). Data visualization, cell counts, and a viability analysis were performed using the CytExpert program.

### 4.7. Immunoblotting

Immunoblotting was performed as previously described [53]. Mouse ESCs or STO cells were trypsinized, collected, and resuspended with Laemmli loading buffer. After 12% or 8% SDS-PAGE electrophoresis proteins were transferred to the nitrocellulose membrane with semi-dry transfer (Helikon, Moscow, Russia), the Ndufs1 and Ndufb10 proteins were detected with rabbit antibodies (PA5-22309, Thermo Fisher Scientific, Waltham, MA, USA) (1:1000 dilution) and (ab196019, Abcam, Cambridge, UK) (1:1000 dilution), respectively. For a loading control, mouse anti-beta-actin antibodies (MA1-744, Thermo Fisher Scientific, Waltham, MA, USA) (1:5000 dilution) were used. Anti-mouse and rabbit HRP-conjugated antibodies were used as secondary antibodies (#115-036-062 and #111-036-045, Jackson ImmunoResearch, West Grove, PA, USA) (1:5000 dilution). Chemiluminescence was detected with ChemiDoc Touch (Bio-Rad Laboratories, Hercules, CA, USA).

### 4.8. Immunocytochemistry

Immunostaining was performed as previously described [50]. Cells were grown in 48-, 24-, or 12-well plates; fixed with 4% paraformaldehyde; and washed and permeabilized with 0.1% Triton X-100. Cells were incubated with primary antibodies: mouse anti-Oct4 (Santa Cruz Biotechnology, Dallas, TX, USA) (1:500 dilution), rabbit anti-Nanog (Bethyl Laboratories, TX, USA) (1:1000 dilution), rabbit anti-SSEA1 (Life Technologies, USA) (1:500 dilution), or rabbit anti-Foxa2 (Santa Cruz Biotechnology, Dallas, TX, USA) (1:500 dilution), which were diluted in PBS, 0.1% Tween20, and 3% bovine serum albumin. After overnight incubation, cells were washed and incubated with secondary antibodies, Alexa647 anti-mouse or Cy3 anti-rabbit antibodies (Jackson ImmunoResearch, West Grove, PA, USA) (1:500 dilution), for 1 h at room temperature. Cells were washed, co-stained with DAPI, and analyzed with an EVOS Cell Imaging System (Thermo Fisher Scientific, Waltham, MA, USA).

### 4.9. Ethics Statement

All animal procedures were performed according to the guidelines for the humane use of laboratory animals, with standards prescribed by the American Physiological Society. The animal procedures were performed at the Institute of Cytology RAS in strict agreement with the animal protection legislation acts of the Russian Federation and were approved by the Animal Welfare Assurance of the Institute of Cytology RAS, Saint Petersburg, Russia, and was received by the Office of Laboratory Animal Welfare, NIH/OD/OER, Bethesda, MD, USA—the Assurance Identification number is F18-00380 (period of validity 12 October 2017–31 October 2022).

### 4.10. Statistical Analysis

Statistical significances of observed differences were evaluated by two-tailed Student’s *t*-test with the use of MS Excel software.

## Data Availability

The row data generated and analyzed during this study are available from the corresponding author.

## Supplementary Materials

The supporting information can be downloaded at: https://www.mdpi.com/article/10.3390/ijms231810924/s1.
