# Supplementary material for: Dual Mode of Mitochondrial ROS Action during Reprogramming to Pluripotency
Source: Int J Mol Sci. 2022 Sep 18;23(18):10924. doi: 10.3390/ijms231810924 (PMC9506067; doi:10.3390/ijms231810924)
Supplement: Supplementary file 1 [file ijms-23-10924-s001.zip › ijms-1909045-supplementary.pdf]

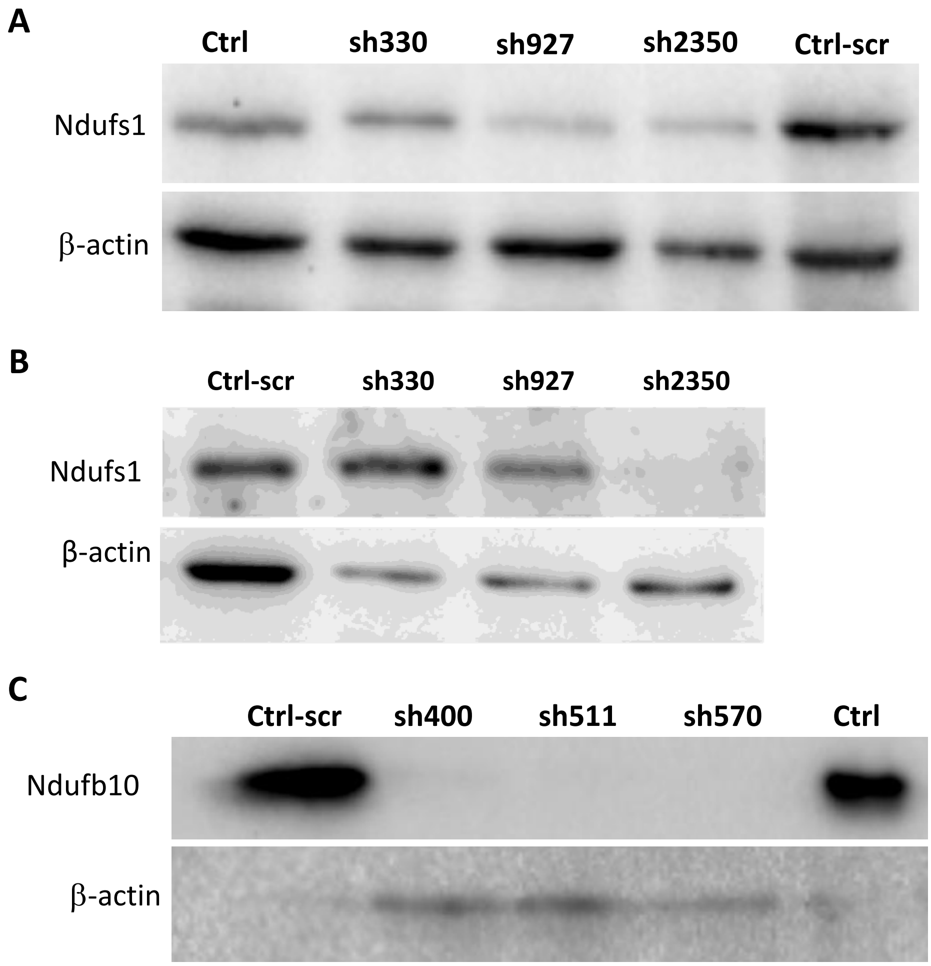

Figure S1. Suppression of Ndufs1 or Ndufb10 expression by shRNA constructs, identified by Western blot. (A) sh330, sh927 and sh2350 shRNAs are significantly suppressed Ndufs1 protein level in STO cells. (B) sh927 and sh2350 shRNAs significantly suppressed Ndufs1 protein level in mouse ESCs (2 weeks selection on puromycin). (C) All shRNAs are completely suppressed Ndufb10 protein level in STO cells.

Figure S2

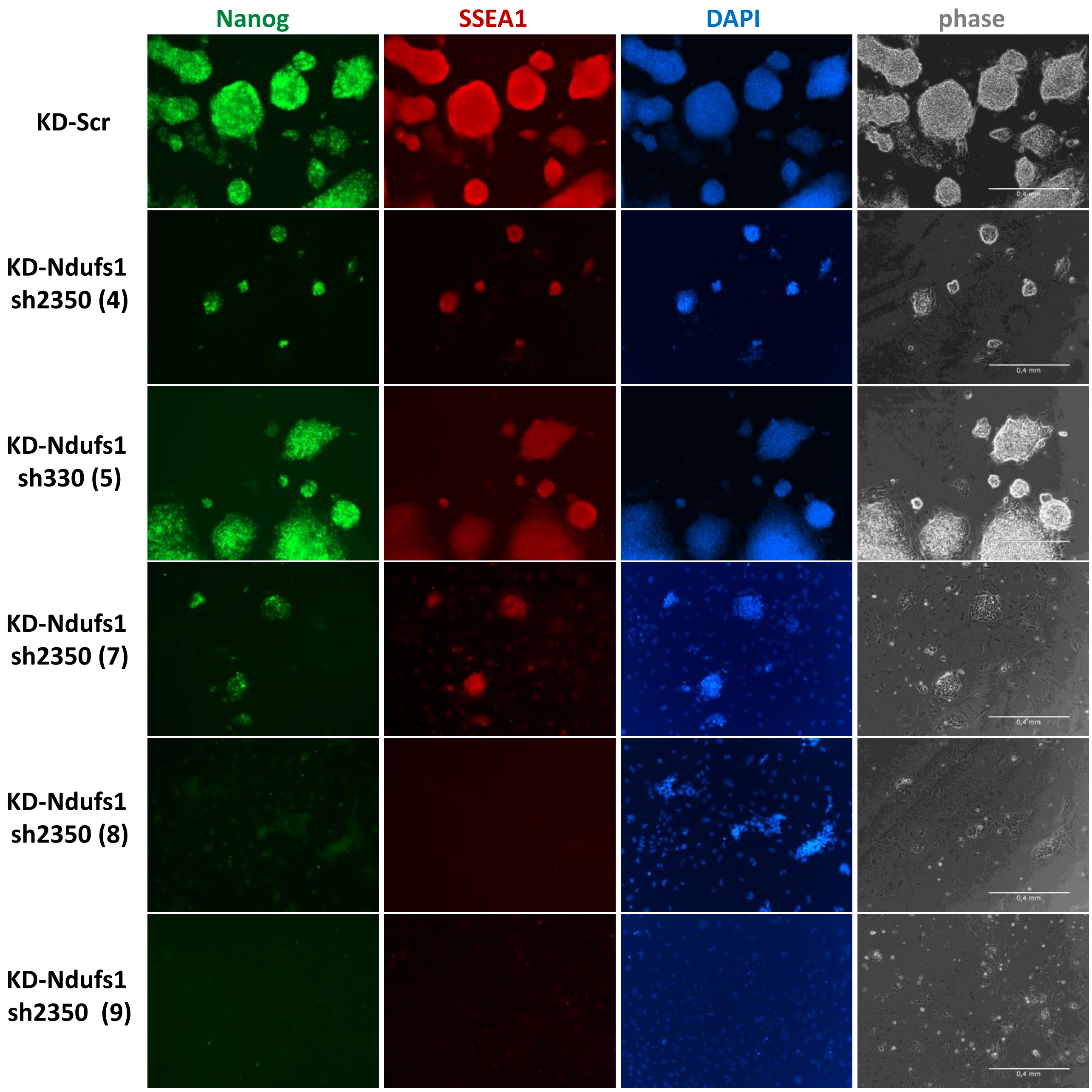

Figure S2. Ndufs1 shRNA knockdown iPSCs clones failed to be maintained and tend to differentiate on 2nd passage after an original clone harvesting. Immunostaining of 2nd passage KD-Ndufs1 iPSC clones for pluripotency markers - Nanog (green), SSEA1 (red) is revealed reduced population of pluripotent cells in clones # 4, and 7, and lack of pluripotent cells in clones # 8 and 9.

Figure S3

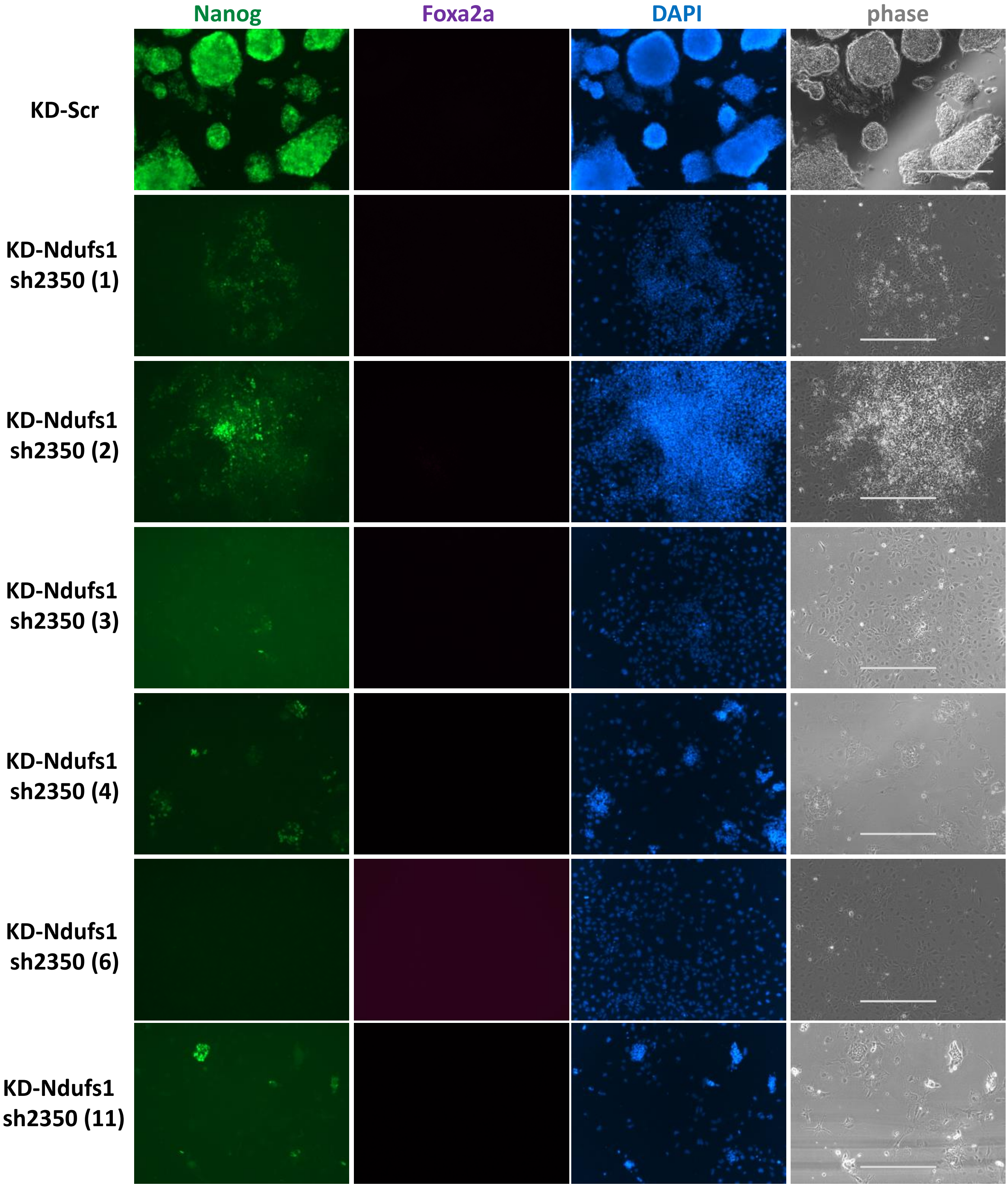

Figure S3. Ndufs1 shRNA knockdown iPSCs clones failed to be maintained and tend to differentiate on 2nd passage after an original clone harvesting. Immunostaining of 2nd passage iPSC clones of KD-*Ndufs1* for pluripotency marker - Nanog (green), and endodermal marker - Foxa2a (purple) revealed reduced population of Nanog+ cells in clones # 1, 2, 3, 4, 11, lack of Nanog+ cells in clone # 6, and luck of any Foxa2a+ cells in all clones.

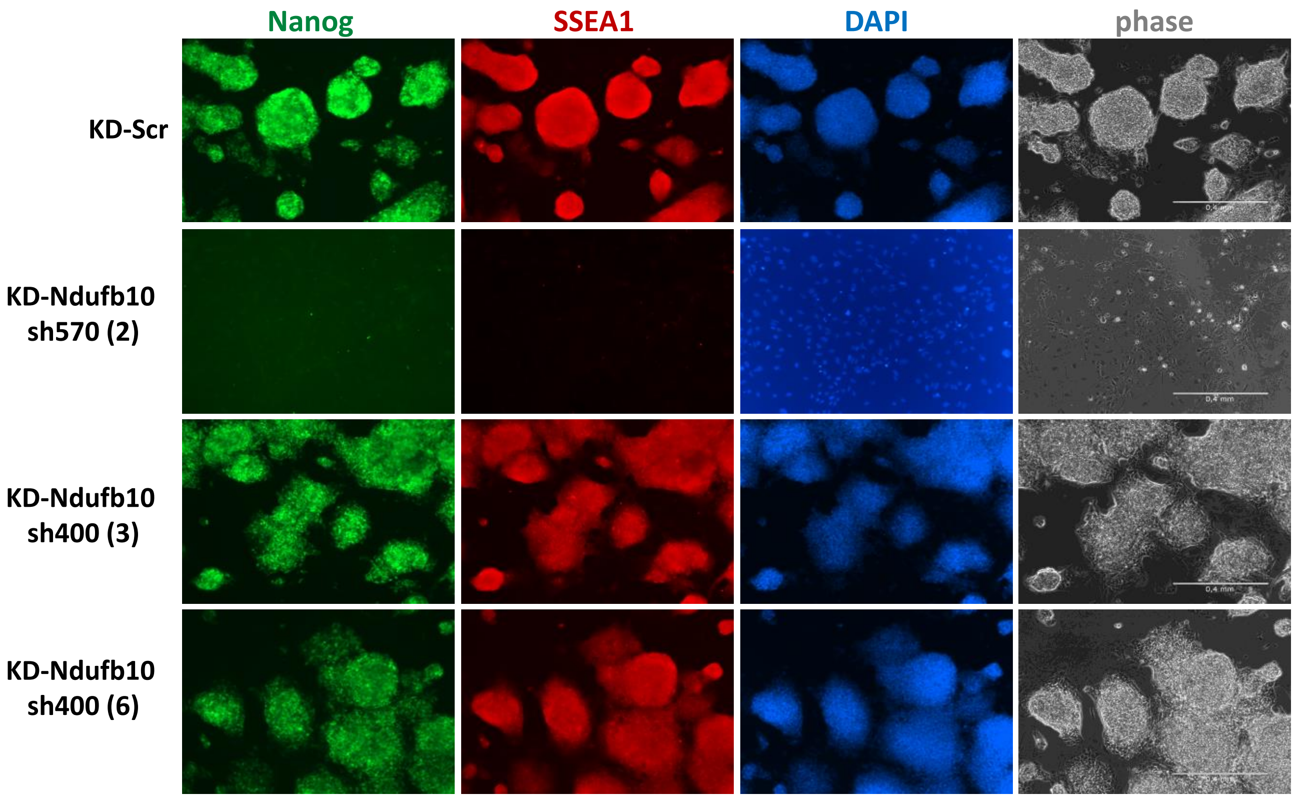

Figure S4. Ndufb10 shRNA knockdown iPSCs clones on 2nd passage after an original clone harvesting. Immunostaining of 2nd passage KD-*Ndufs1* iPSC clones for pluripotency markers - Nanog (green), SSEA1 (red) revealed lack of pluripotent marker positive cells in clone # 2.

Figure S5

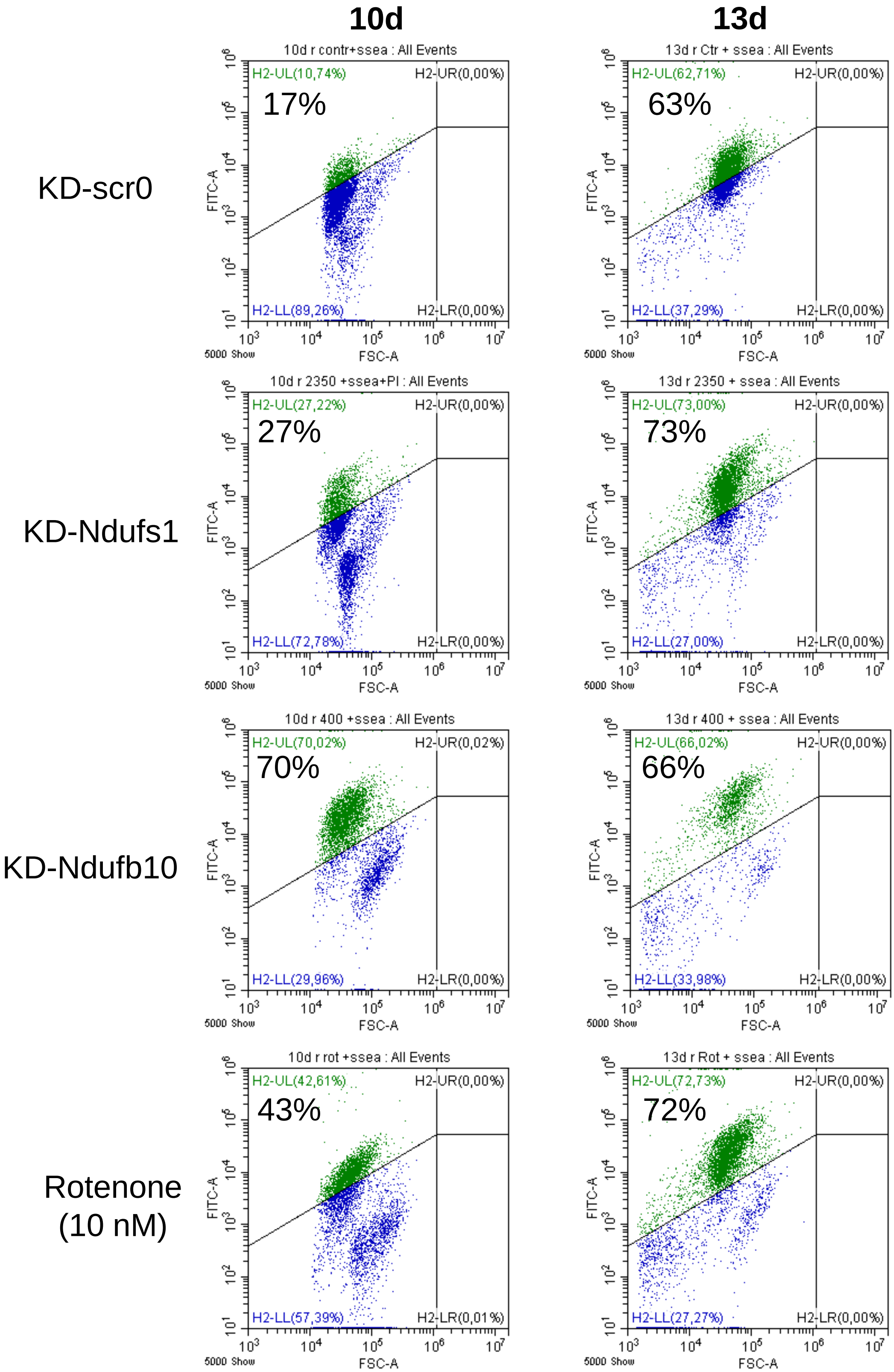

Figure S5. Increase in number of SSEA1-positive cells at day 10 upon inactivation of NDUFS1 or NDUFB10 or treatment with rotenone during entire reprogramming process. FACS profiles of cell immunofluorescence staining with anti-SSEA1 antibody indicated increase in number of SSEA1-positive cells at day 10, not at day 13.

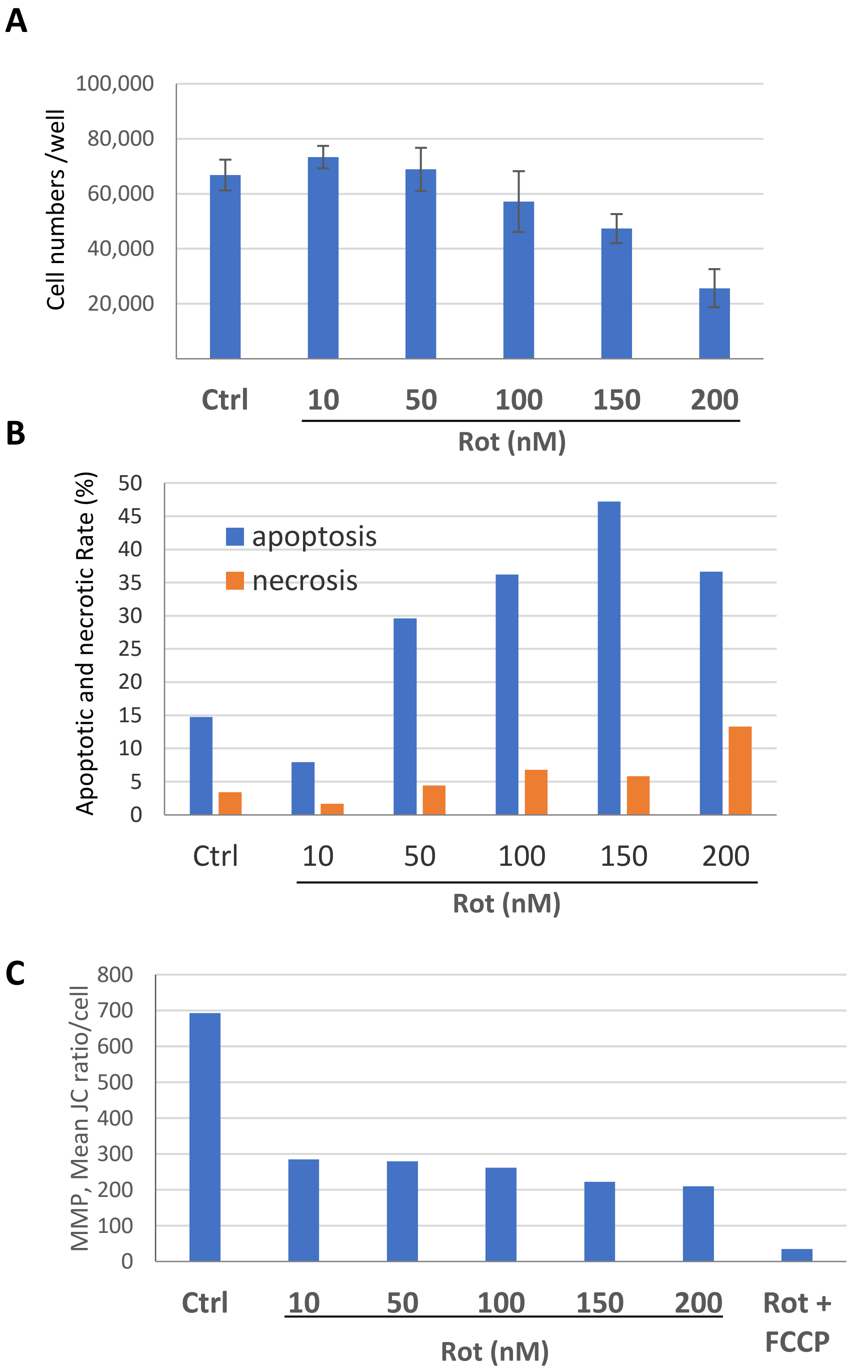

Figure S6. Rotenone concentrations that affected mitochondrial membrane potential (MMP), and does not affect viability and apoptosis of iPSCs. (A) Rotenone in concentration 10-50nM (X axis) does not affect proliferation of iPSCs. (B) Rotenone (10nM) does not induces apoptosis of iPSCs, however at concentrations starting from 50nM (X axis) are induced apoptosis in iPSCs. (C) FACS assay JC-1 staining of iPSCs treated by rotenone (*x* axis – Rot concentrations, nM) is revealed a robust MMP reduction. FCCP upon rotenone and FCCP tre.

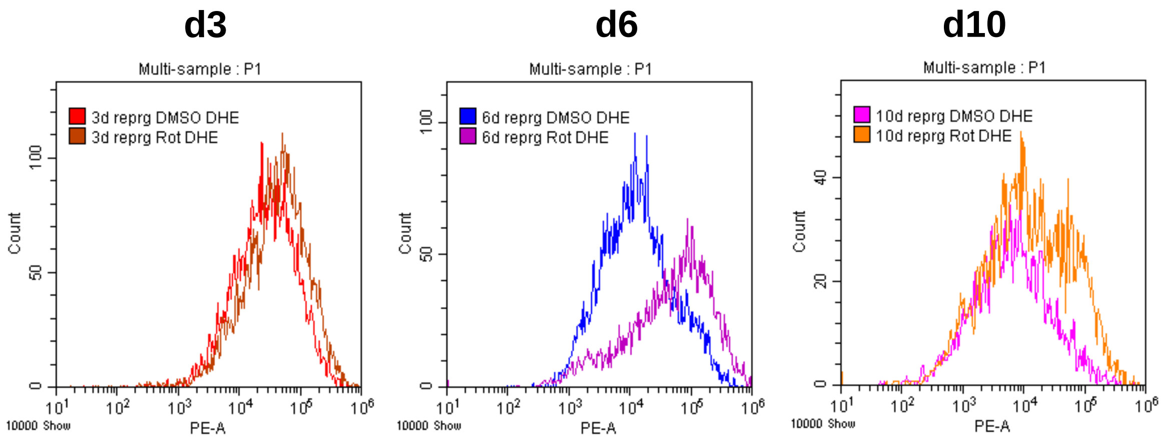

Figure S7. Increased ROS generation by rotenone during different stages of cell reprogramming. FACS profiles of cell fluorescence revealed by DHE staining is indicated an increase in ROS generation (DHE staining) by rotenone (10nM) treatment during day 3, 6 and 10 of the process if compared to vehicle (DMSO) treated cells.

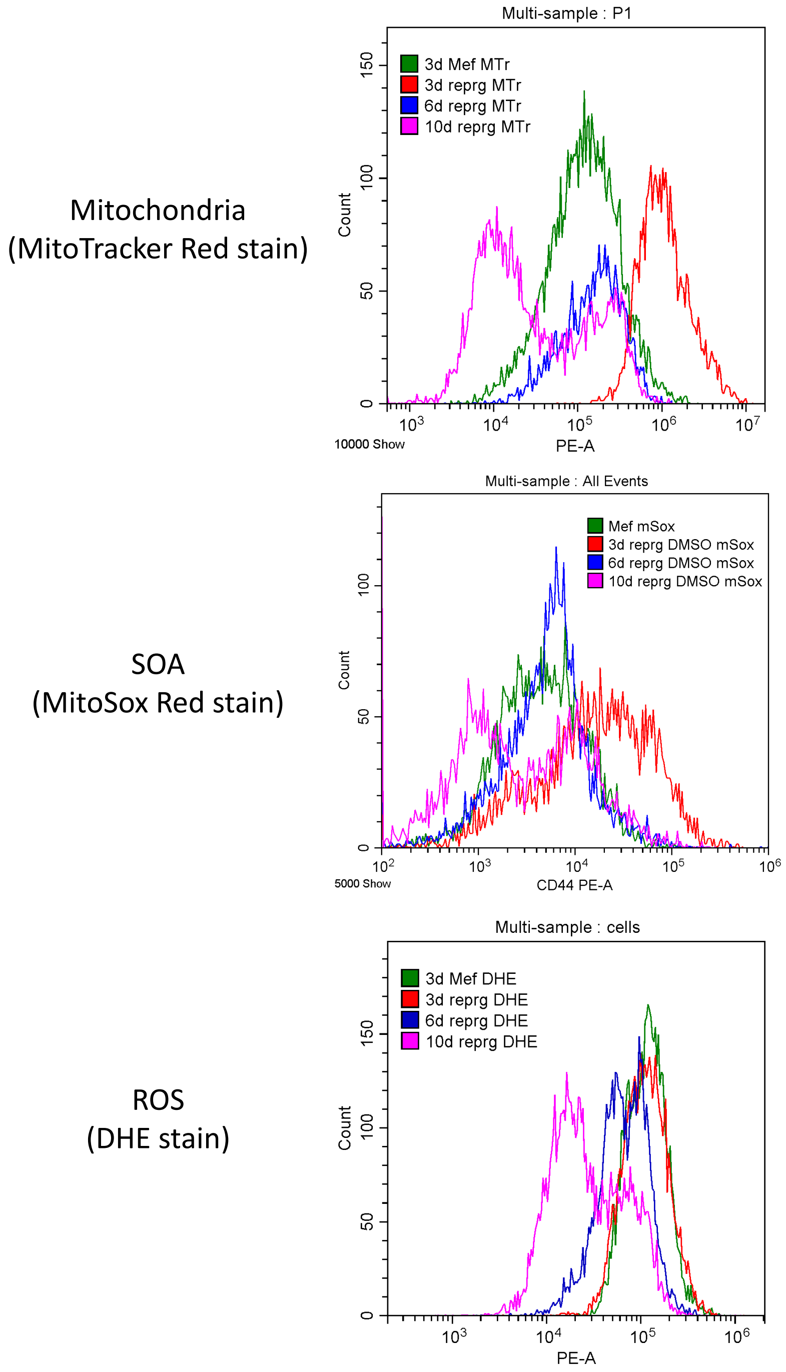

Figure S8. Reduction in mitochondria content and ROS generation during cell reprogramming process. FACS profiles of cell fluorescence revealed by MitoTracker Red, MitoTracker Red, and DHE staining indicated decrease number of mitochondria, in level of SOA and ROS from day 3 to 6th and 10th days of cell reprogramming to pluripotent state.

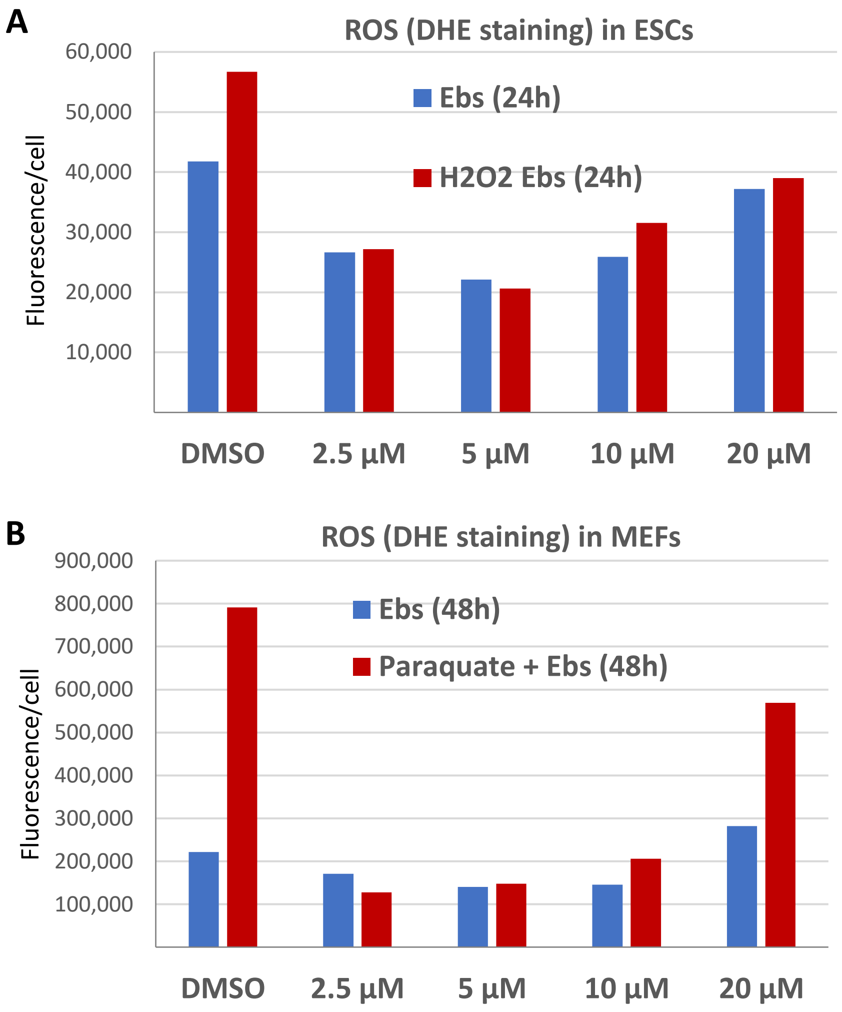

Figure S9. Determination of optimal working concentrations of Ebselen. DHE intensity in ESCs (A) and MEFs (B) is determined by FACS assay by the induction of ROS with hydrogen peroxide (A) or paraquat treatments (B). y-axis indicates mean DHE fluorescence per cell.

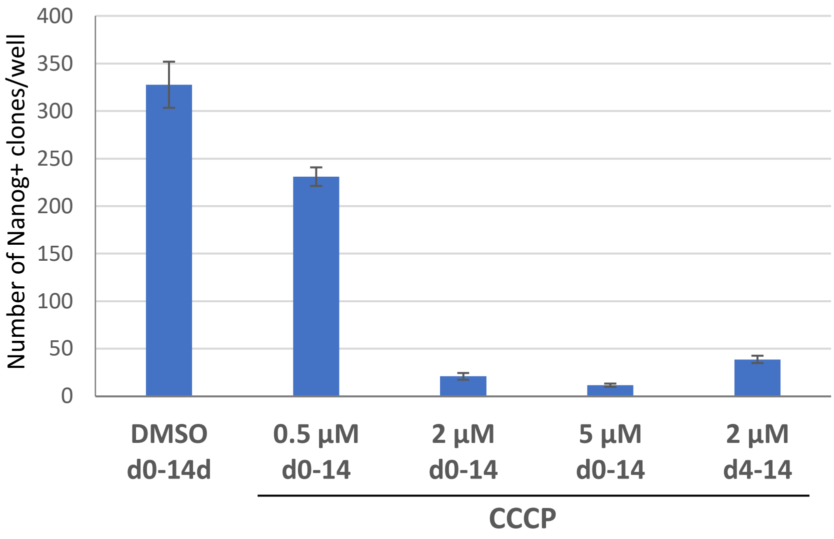

Figure S10. Mitochondrial function are important for the efficient reprogramming of MEFs into iPSCs. Attenuating mitochondrial function with at 0.5, 2, and 5 µM CCCP during all stages of the reprogramming leads to a strong suppression of the process (n=3, P<0.0001). Attenuating mitochondrial function by CCCP at 2 µM during days 4-14 of the reprogramming leads to a strong suppression of the process (n=3, P<0.0001). The y-axes indicates the number of Nanog-positive clones on day 14 of reprogramming ±SD.
